# Long-Term Effects of Multiple-Micronutrient Supplementation During Pregnancy, Lactation, and Early Childhood on the Cognitive Development of Children Aged 4–14 Years: A Systematic Review of Randomized Controlled Trials

**DOI:** 10.3390/nu17243966

**Published:** 2025-12-18

**Authors:** Arnold William, Carl Lachat, Dimitrios Petalios, Alice Deshons, Kokeb Tesfamariam Hadush, Mélanie Broin, Souheila Abbeddou

**Affiliations:** 1 Department of Food Technology, Safety, and Health, Faculty of Bioscience Engineering, Ghent University, 9000 Ghent, Belgium; williamarnoldj@gmail.com (A.W.); carl.lachat@ugent.be (C.L.); dimitrios.petalios@gmail.com (D.P.); 2 Department of Health System, Policy and Impact Evaluations, Ifakara Health Institute (IHI), Dar es Salaam P.O. Box 78373, Tanzania; 3 Agropolis International, F-34094 Montpellier, France; alice.deshons@gmail.com (A.D.); broin@agropolis.fr (M.B.); 4 Department of Bioanalysis, Faculty of Pharmaceutical Sciences, Ghent University, 9000 Ghent, Belgium; kokebtesfamariam.hadush@ugent.be; 5 College of Medicine and Health Sciences, Arba Minch University, Arba Minch P.O. Box 21, Ethiopia; 6 IMCAN (Infant, Maternal, Child, and Adolescent Nutrition), 9000 Ghent, Belgium; 7 Department of Public Health and Primary Care, Faculty of Medicine and Health Sciences, Ghent University, 9000 Ghent, Belgium

**Keywords:** multiple-micronutrient supplements, point-of-use micronutrient powder, pregnancy and lactation, early childhood, cognitive development, systematic review

## Abstract

**Background:** Inadequate nutrition, poor health care, and limited stimulation constrain early childhood development and cognitive potential. Micronutrient deficiencies during pregnancy and early life are prevalent in low- and middle-income countries (LMICs) and may impair cognitive outcomes. Maternal multiple-micronutrient (MMN) and point-of-use micronutrient powder (MNP) supplements improve birth outcomes and iron status, but their long-term cognitive impact remains unclear. This systematic review assessed the long-term impact of maternal MMN and early-childhood MNP supplementation on cognitive development among children aged 4–14 years in LMICs. **Method:** Following PRISMA guidelines (PROSPERO CRD42023459846), (cluster) randomized controlled trials were identified from six databases and gray literature (October 2023; updated July 2025). Records were managed in EndNote and screened in Covidence, and data were synthesized using Review Manager. Eligible studies examined MMN or MNP interventions during pregnancy, lactation, or early childhood, reporting cognitive, motor, or socio-emotional outcomes in children aged 4–14. **Results:** Ten studies met the inclusion criteria: six on maternal supplementation, three on early childhood interventions, and one combining both. Most were conducted in Asia, with one in Tanzania and one in Peru. Although most findings were not statistically significant, two large UNIMMAP-based trials indicated modest long-term improvements in procedural memory and intelligence, while one early childhood point-of-use MNP trial suggested enhanced pre-academic skills. **Conclusions:** Maternal MMN supplementation may modestly enhance specific domains of cognitive development, whereas evidence on the long-term effects of MMN and point-of-use MNPs on cognitive development remain limited, highlighting the necessity for further research.

## 1. Introduction

Micronutrients are essential vitamins and minerals required in the body in small quantities to ensure healthy growth and development [1]. The requirement for micronutrients increases during pregnancy, the postpartum period, and early life, a time that coincides with the brain development in the fetus and young child, forming the foundation for cognitive development [2,3,4]. Neurodevelopment unfolds dynamically within the first two to three weeks after conception, when the neural tube initiates cell division to give rise to neurons and supportive glial cells [5].

Globally, approximately 40% of women of reproductive age have inadequate micronutrients intake, with 42% of pregnant women suffering from iron-deficiency anemia [6,7]. Maternal micronutrient deficiencies in early pregnancy can disrupt essential processes, such as cell proliferation and differentiation, which are critical for brain development and cognitive function in children [8]. These deficiencies can also impair key developmental stages, including synapse formation, dendrite branching, and neural tube development, thereby impacting cognitive outcomes [9]. Furthermore, over 50% of children under 5 years of age suffer from micronutrient deficiencies worldwide, with a large proportion (approximately 42%) residing in Africa and Asia [1]. During infancy and early childhood, deficiencies in essential nutrients such as iron, folic acid, zinc, iodine, and vitamin A have been associated with adverse health outcomes in children, including anemia, wasting, and developmental delays [8].

Micronutrient deficiencies are more prevalent in low- and middle-income countries (LMICs), where dietary diversity and food quality are often limited [10]. In response, the World Health Organization (WHO) has established global recommendations for supplementation, fortification, and dietary diversification, to prevent micronutrient deficiencies during pregnancy, lactation, and early childhood [11]. There is evidence to support the effect of single micronutrients on child development. For example, taking 360–4000 µg of folic acid per day before conception to 12 weeks of pregnancy can prevent 40–80% of neural tube defects [12,13,14]. Iron supports neurodevelopment, myelination, and neurotransmission in the brain, as well as developmental processes. Iron deficiency in early life can result in long-lasting or permanent neurocognitive and behavioral disorders [15].

The WHO recommends daily supplementation with 30–60 mg of elemental iron and 400 µg of folic acid during pregnancy and postpartum [16]. Iron and folic acid (IFA) supplementation prevents maternal anemia, puerperal sepsis, low birth weight, and preterm birth in settings where anemia affects more than 20% of the population. Furthermore, zinc and calcium are also recommended in certain contexts [16,17]. As micronutrient deficiencies often coexist, particularly in LMICs, the 2020 updated guideline recommends multiple-micronutrient (MMN) supplementation, including IFA during pregnancy and the postnatal period [8,18]. The WHO recommends point-of-use fortification of complementary foods for infants and young children (6–23 months) and foods for children aged 2–12 years with iron-containing micronutrient powders (MNPs) to improve iron status and reduce anemia [19].

The available evidence suggests that maternal MMN supplementation has a positive short-term effect on children’s cognitive development [8,20,21,22,23]. However, evidence on the effects of MNPs on young children’s cognitive development is mixed. For example, in a retrospective cohort study, Geletu et al. found that providing low-iron MNPs to children aged 6–9 months for three months reduced anemia and stunting, and improved motor development at 9–12 months [22]. In contrast, Luo et al. reported that providing MNPs to children aged 6–11 months for 18 months had no significant effect on anemia or cognitive outcomes at the end of the intervention [24]. Furthermore, there is little evidence on the long-term effects in promoting cognitive development, and no systematic summary has been conducted of the trials investigating the long-term impact of maternal MMN and child’s MNP supplementation on children’s cognitive development in LMICs. Therefore, in response to the evidence needs prioritization exercise conducted by the Nutrition Research Facility (NRF) in consultation with decision-makers in Asia, the question of the long-term impact of maternal MMN supplementation and early childhood point-of-use MNPs on cognitive development in LMICs was identified as a top priority for nutrition programming during a virtual regional workshop held on 19–20 April 2022.

Given the lack of systematic synthesis of long-term cognitive outcomes following MMN and MNP interventions, this review aimed to evaluate the evidence for their sustained effects on cognitive development among children aged 4–14 years in LMICs.

## 2. Methods

This systematic review adhered to the Preferred Reporting Items for Systematic Reviews and Meta-Analyses (PRISMA) (Supplementary Table S1 Checklist) [25]. We registered the protocol on PROSPERO (CRD42023459846) and published it on 26 September 2023, then updated it on 2 June 2025. We conducted the initial literature search on 26 October 2023, and updated the search on 22 July 2025.

### 2.1. Data Sources and Search Strategies

The search strategy was developed for MEDLINE (PubMed) and then adapted for five other databases, namely EMBASE, Cochrane (CENTRAL), Web of Science, CINAHL, and Scopus. Gray literature was also searched via Google Scholar. Only peer-reviewed articles, while unpublished data, abstracts, reports, and conference proceedings were excluded. Full search strategies and key terms are reported in Table S2.

### 2.2. Eligibility Criteria

This systematic review included randomized controlled trials (RCTs) or cluster-RCTs. References cited in systematic reviews and meta-analyses were also screened for relevant additional records. We included studies if (1) the study supplemented population was—women, pregnant or lactating, or—infants and young children (from birth to 3 years of age), (2) the intervention group received MMN that was defined as at least containing three micronutrients [26], (3) the outcomes assessed in children 4–14 years of age, (4) were conducted in LMICs as classified by the World Bank [27], and (5) assessed at least one of the development domains in children: intelligence, memory, concentration, psychomotor skills, and academic achievement, social-emotional development, and adaptive skills. There was no restriction regarding the duration of the intervention, language and the year of publication. Studies limited to children under 4 years of age, or those with developmental disability, including cretinism, were excluded. Comparators included IFA, folic acid alone, placebo or no supplement. In multi-arm studies, IFA was considered the standard for maternal interventions and the placebo for children’s supplementation interventions.

### 2.3. Outcome Measures

The primary outcome of interest was the cognitive development of children aged 4–14 years whose mothers received MMNs during pregnancy or lactation, as well as infants and young children (6 months–3 years) who received MNPs. Cognitive development outcomes were assessed across multiple domains, including general cognitive performance, language and communication, motor skills, social-emotional development, adaptive behavior, memory, executive functioning, and educational attainment [20]. Validated instruments capturing these domains, such as measures of attention, memory, learning, verbal and non-verbal language, and processing speed, were used across the included studies. A summary of standardized tools employed for cognitive assessment is presented in Table S3, adapted from Prado et al. and updated to include additional instrument identified in this review by A.W. [28].

### 2.4. Study Selection and Screening

Records identified using the search strategy from the respective databases were exported to EndNote X20 (www.endnote.com). All records were then exported to Covidence systematic review software (Veritas Health Innovation, Melbourne, Australia. Available at www.covidence.org), to identify and remove duplicates, and conduct a screening based on the title and abstract, and then of the full text. Two reviewers (A.W. and S.A.) independently screened titles, abstracts, and full-text articles in Covidence, resolving any disagreements through discussion or, when needed, consultation with a third reviewer.

### 2.5. Data Extraction

Data extraction was performed independently by two reviewers (A.W., S.A.) using a modified Cochrane data collection form (https://dplp.cochrane.org/data, accessed on 29 November 2023), tailored to the research question. The intervention compared MMN supplementation with control groups of pregnant or lactating women, and point-of-use MNPs for infants or young children (6 months–3 years) who received a placebo, no supplement, or standard care (such as folic acid or IFA). This design allowed assessment of the long-term effects of MMN and MNPs on cognitive outcomes in children aged 4–14 years. Data extraction captured study design, participant characteristics, intervention details, follow-up duration, and cognitive outcome measures.

### 2.6. Assessment of Risk of Bias

The risk of bias (RoB) was assessed using the Cochrane risk of bias assessment tool (RoB2) (https://sites.google.com/site/riskofbiastool/welcome/rob-2-0-tool, accessed on 14 January 2024) to ensure the validity and reliability of findings. The assessment covered five key domains: (1) randomization process, (2) intervention deviation, (3) missing outcome data (4) measurement of the outcome, and (5) selection of the reported result. Two reviewers (A.W. and S.A.) conducted these assessments, resolving disagreements through discussion or, if necessary, consultation with a third reviewer (C.L.).

### 2.7. Data Synthesis

Data were handled using Review Manager (RevMan version 5.4). Given the variability in assessment tools and the limited number of studies per outcome, a qualitative synthesis was conducted. Main results were analyzed by outcome type and study, with effect sizes expressed as standard mean differences (SMDs) and 95% confidence intervals (95%CI). Pooled SMDs were calculated using a random-effects model to account for between-study variability [29]. When standard deviations (SD) were not provided, they were derived from the 95%CI and sample size. Multi-arm trials were analyzed separately to preserve the distinction between intervention effects. Control groups included IFA for maternal interventions and placebo for child interventions. Due substantial heterogeneity, meta-analysis was not performed. Instead, findings were summarized using vote counting based on the direction of effect across cognitive, motor, and behavioral outcomes. A sign test (binomial probability test) was applied to assess the overall direction of effects across studies within each outcome domain (GraphPad, two-tailed *p*-value).

## 3. Results

Six databases were searched on 26 October 2023, yielding 12,284 records. After removing 3469 duplicates using Covidence systematic review software, 8815 records were excluded based on title and abstract screening. A total of 74 full-text articles were assessed for eligibility, of which 64 were excluded, leaving 10 studies on maternal and child supplementation included in this review. An updated search conducted on 22 July 2025, identified an additional 1971 records. Following screening, no new studies met the eligibility criteria, and the original 10 studies remained in the final set of included articles.

**Figure 1:**
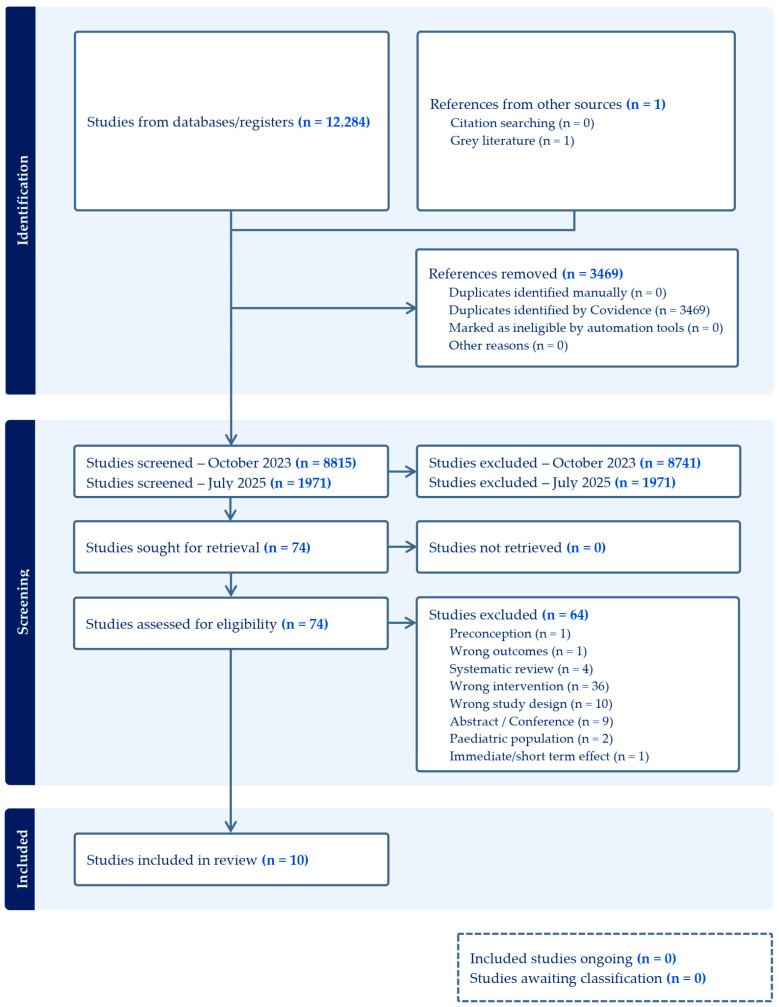
Effect of micronutrient supplementation during pregnancy and lactation, and early childhood on long-term development of children.

In accordance with PRISMA guidelines, the study selection process is summarized in Figure 1, which details the reasons for study inclusion and exclusion.

### 3.1. Characteristics of Supplementation Intervention Trials

These characteristics refer to the original parent studies from which eligible follow-up investigations were derived (Table 1). Most of the parent trials were conducted in Asia, with two based in Tanzania [30,31], and one in Peru [32,33]. Study designs included RCTs [30,31,33,34] and cluster-RCTs [35,36,37,38,39,40], with sample sizes ranging from 242 to 31,290 participants.

Three parent trials targeted infants and young children aged 1–36 months [31,38,39,40], providing IFAZn in Nepal [38,39], multiple vitamins with or without zinc in Tanzania [31], or point-of-use sprinkles in Pakistan [40]. Control groups received placebo [31,38,39] or no supplementation [40].

Among the parent trials enrolling pregnant women, gestational age at enrollment ranged from conception to 28 weeks, with supplementation typically continuing until delivery, or extended into the postpartum period (1 month [33], 6 weeks [30], and 90 days [35,36]). Maternal baseline BMI ranged from 19.0 to 23.6 kg/m^2^, and several populations showed a high prevalence of undernutrition and anemia. Reported adherence ranged from 73% to 96%, although not all parent studies provided compliance data [30,33,34].

Six studies supplemented pregnant women [30,33,34,35,36,37], using IFA plus zinc (IFAZn) [33,35], multiple vitamins [30], or MMN formulations [34,35,36,37]. Control groups received IFA [30,33,34,35,36,37], vitamin A [35], or FA [35,37]. Three of the MMN formulations followed the United Nations International Multiple Micronutrient Antenatal Preparation formulation (UNIMMAP 13–15 micronutrients) [34,36,37], though minor variations existed (e.g., lower vitamin B12 in Shankar et al. [36], higher vitamins A, B1, B2, B3, B6, and C in Christian et al. [35]). Folic acid content ranged from 400 to 800 µg (Table 2).

### 3.2. Risk of Bias in Included Studies

Table 3 provides a summary of the risk-of-bias assessment across multiple domains, as evaluated using the Cochrane RoB2 tool. Seven studies were rated as low risk, while two were judged to have some concerns [43,44] and one as high risk [45]. In Christian et al., one maternal supplementation group (folic acid only) was excluded from follow-up analyses, as the authors reported no effect on child cognitive outcomes [43]. In Sudfeld et al., outcomes for children receiving multiple vitamins with or without zinc were compared against placebo or zinc alone, but results were not reported by individual group [44]. Christian et al. included only offspring from two of the five original maternal supplementation groups, both with high dropout rates and baseline imbalances between participants [45]. These limitations were considered to have affected the overall quality of the evidence.

### 3.3. Characteristics of the Follow-Up Studies

Included studies explored the long-term effects of MMN supplementation and MNPs interventions during pregnancy, lactation, and early childhood on cognitive and developmental outcomes in children aged of 4–14 years (Table 4). All studies reported on cognitive development, five on motor development [28,43,45,50,51], and five studies on behavioral development and mental health [28,44,46,49,50].

Follow-up periods varied, with some studies conducting repeated assessments. This review reports outcomes at the latest eligible time point. For example, in China, supplementation effects were assessed at 1 year [52], 7–10 years [53], and 10–14 years, with only the latter included here [48,49]. Prado et al. examined child development at both 42 months [23] and 9–12 years [28], with only the latter retained. The included studies also varied considerably in terms of sample size, ranging from 184 to 2879 participants.

### 3.4. Long-Term Effects of Multiple-Micronutrient Supplementation on Cognitive Outcomes

#### 3.4.1. Maternal Supplementation

Six studies evaluated the long-term effects of maternal MMN supplementation on child and adolescent cognitive development (Table 4).

General intelligence and executive function. Four analyses compared MMN to IFA. Zhu et al. reported improved intelligence at ages 10–14 years [48,49], whereas Christian et al. observed reduced performance intelligence and no benefit, or a decline, in executive function at ages 7–9 years [43]. Dulal et al. found no effects on cognition at 12 years [47]. Prado et al. similarly observed no significant effect on intelligence, declarative memory, executive function, or academic achievement at ages 9–12 years, though procedural memory improved, and positive but non-significant trends favored MMN across outcomes [28]. In Tanzania, Sudfeld et al. reported no differences between MMN and placebo on intelligence or executive function at 11–14 years [44].

Two additional studies compared IFAZn to IFA. Caulfield et al. found no effects on intelligence at 54 months [46], and Christian et al. reported no benefit on intelligence at 7–9 years and mixed effects on executive function [43].

Motor development. Three studies reported on motor outcomes. Prado et al. found no effect on fine motor dexterity [28]. By contrast, Christian et al. observed poorer motor performance, on the *MABC* and *finger-tapping* tests, among children exposed to maternal IFAZn or MMN compared with IFA [43]. Christian et al. suggested that zinc co-supplementation may have attenuated iron’s beneficial effect on motor outcomes, possibly due to competitive inhibition of absorption [54,55].

Socio-emotional development. All available studies consistently found no effect of maternal MMN supplementation on socio-emotional outcomes, irrespective of sample size [28,44,46,49].

#### 3.4.2. Infant and Young Child Supplementation

Three studies assessed MMN supplementation or point-of-use fortification with MNPs in early childhood (Table 1). In Nepal, Murray-Kolb et al. reported no effects of preschool IFAZn supplementation on intellectual, executive, or motor function at 7–9 years, aside from a positive effect on Stroop test performance [51]. In Tanzania, 18 months of supplementation with multivitamins containing vitamins B-complex, C, and E, with or without 5 mg of zinc, showed no greater effects on intelligence, executive function, or mental health at ages 6–8 years compared with zinc alone or placebo [44]. In Pakistan, use of MNPs from 6–24 months had no effect on cognition, motor, or socio-emotional outcomes at 4 years, although pre-academic skills improved relative to non-supplemented children, and responsive stimulation had a greater effect size [50].

Overall, these findings suggest no consistent long-term developmental benefits of MMN supplementation or MNP use in early childhood, though evidence remains limited and inconclusive.

#### 3.4.3. Combined Maternal and Child Supplementation

One study examined combined maternal and child IFAZn supplementation [45]. At ages 7–9 years, children in the combined supplementation group performed worse on backward digit span, motor tasks (MABC, finger-tapping), and the *Stroop test*, though intelligence scores (UNIT) were unaffected.

#### 3.4.4. Effect Direction Results for Developmental Outcomes in the Included Studies

Table 5 presents the effect direction plot for cognitive, motor, and behavioral development outcomes across the 10 included studies. For cognitive development, one study demonstrated a positive effect, none reported a negative effect, and all showed mixed or conflicting results across at least one cognitive domain. For motor development, three studies reported negative effects, while another three reported conflicting or unclear results; no study demonstrated a positive effect. The sign test for motor outcomes yielded a *p*-value of 0.25. For behavioral development, six studies reported conflicting or unclear findings, while one study identified a positive effect.

## 4. Discussion

The evidence from this systematic review which investigated the potential long-term effects of MMN supplementation during pregnancy and lactation, as well as point-of-use fortification with MNPs during early childhood, on children’s cognitive and developmental outcomes in LMICs remains inconsistent and limited in quality.

Across ten (cluster) randomized controlled trials, the interventions were evaluated in children aged 4–14 years. The findings suggest that maternal MMN supplementation may contribute to small improvements in certain/selected cognitive outcomes, while the evidence for long-term effects of MNPs in early childhood remains limited and inconsistent. Variation in results across studies likely reflects differences in study design, baseline nutritional status, environmental exposures, timing and frequency of follow-up assessments, and methodological inconsistencies in cognitive testing.

Evidence from observational studies of single nutrients, such as iron, has suggested potential cognitive benefits. However, the absence of strong and consistent effects of MMN supplementation may reflect a “dilution effect,” where benefits are masked by other determinants of child development, including adequate dietary intake, illness, medications, and psychosocial stimulation [56]. While many individual studies reported non-significant findings, the overall trend indicates modest improvements in cognitive outcomes for children whose mothers received MMNs, suggesting that these effects are unlikely to be explained by chance alone [28]. For example, Prado et al. observed improvements in procedural memory, whereas Zhu et al. reported small gains in full-scale Intelligence Quotient (IQ) and working memory at ages 10–14 years [48,49]. Conversely, Prado et al. also reported no significant effects on intelligence, declarative memory, executive function, or academic achievement at ages 9–12 years [28], and Sudfeld et al. found no significant improvements in intelligence, executive function, or mental health [44].

Postnatal interventions with MNPs similarly show mixed findings. A trial in Pakistan providing MNPs from 6–24 months reported no long-term effects on intelligence, executive function, motor development, or socio-emotional outcomes at 4 years, although pre-academic skills improved, particularly when nutrition interventions were paired with responsive stimulation [50]. One study of continuous supplementation with IFAZn from pregnancy through early childhood found mixed results at 7–9 years: children showed slightly lower performance on backward digit span and finger tapping tasks but higher scores on motor coordination and Stroop tasks, with no difference in general intelligence [45]. As this remains the only study examining sustained maternal-child supplementation, evidence is insufficient to draw firm conclusions about the long-term cognitive benefits of continuous interventions.

Long-term effects of prenatal supplementation with individual nutrients have also been limited. For example, supplementation with *n*-3 long-chain fatty acids showed no significant effect on IQ at 7 years [57], likely because cognitive development by this age is influenced by multiple factors, including home stimulation, schooling, illness, and diet. Psychosocial stimulation within the home has consistently been shown to exert a stronger effect on child cognition than nutrition interventions alone [28,58,59]. Moreover, subgroup analyses suggest that MMN supplementation may be more beneficial for children of mothers with micronutrient deficiencies or anemia, with population-level effects diluted and not statistically significant [28].

Systematic reviews and meta-analyses support the notion of modest benefits. One review of prenatal and postnatal interventions found that combined micro- and macronutrient supplementation had small effects on cognitive development in children under 2 years, compared with single-nutrient interventions [21,60]. Recent trials since Leung et al. (2011) further suggest that maternal MMN supplementation may have small long-term benefits, though findings are inconsistent and rarely statistically significant [21,46]. A meta-analysis of 20 trials (1970–2008) that assessed the effects of supplementing children with at least three micronutrients compared to placebo found slight improvements in fluid intelligence, reflecting reasoning ability and neurological potential, but no effect on crystallized intelligence, which reflects acquired knowledge [61,62,63].

Overall, maternal MMN supplementation and early childhood MNP interventions may support modest improvements in specific cognitive domains, including procedural memory, working memory, and pre-academic skills. However, effects are inconsistent across studies and populations, and are often overshadowed by environmental, educational, and social influences. Moreover, the available evidence is judged to have some concerns, which reduces the overall certainty of the findings. Given the limited number of long-term trials, particularly those evaluating continuous maternal-child supplementation, definitive conclusions cannot yet be drawn. Further high-quality research is needed to determine whether sustained interventions across the maternal-child continuum can produce measurable cognitive benefits into later childhood and adolescence.

### Limitations and Future Research Recommendations

Most included studies were not specifically powered to assess long-term cognitive outcomes. Heterogeneity in assessment tools, timing of evaluations, and active comparators, such as IFA supplementation, may have diluted intervention effects. Baseline micronutrient status and dietary intakes were often unreported, and socio-environmental factors were rarely considered, despite their strong influence on cognitive outcomes. Ultimately the methodological limitation of the primary evidence limits our study to draw a firm conclusion on the long-term effects of MMN and MNPs on cognitive development of children.

Future research should prioritize larger, long-term RCTs assessing continuous maternal-child interventions with standardized cognitive tools in LMICs settings. Evaluating continuous maternal-child supplementation programs and exploring the mechanisms underlying both positive and negative effects of MMN supplementation are also warranted. Additionally, the interactions between nutrition, socio-emotional stimulation, and education should be examined to optimize cognitive outcomes.

## 5. Conclusions

Maternal MMN supplementation during pregnancy and lactation may have modest long-term effects on cognitive development, while point-of-use MNP fortification in early childhood shows limited evidence for cognitive benefits. Observed trends suggest potential positive effects, but evidence is insufficient to support formal public health recommendations aimed solely at improving long-term cognitive outcomes. Evidence for other health benefits, including improved birth outcomes and reduction of anemia, remains strong, underscoring the importance of continued supplementation programs. Future adequately powered trials with multiple follow-up assessments are needed to clarify the long-term effects of MMN and MNP interventions on cognitive development.

### Public Health Recommendations

While the evidence for long-term cognitive benefits of MMN supplementation and point-of-use MNP fortification remains limited, strong evidence supports other health benefits. Maternal MMN supplementation improves birth outcomes, reducing the risk of low birth weight and small-for-gestational-age births. Point-of-use MNPs effectively improve iron status and reduce anemia in infants and young children. Accordingly, WHO recommendations to replace IFA with MMN during pregnancy and lactation and to use iron-containing MNPs for children aged 6–23 months and 2–12 years should remain unchanged.

Sustainable approaches, including the use of local nutrient-rich foods and fortified products, should be promoted, particularly in contexts where supplementation programs rely on external funding. Combining nutrition with psychosocial stimulation and education likely yields stronger developmental outcomes than nutrition alone. This is essential to maximize developmental outcomes and support broader goals, including Sustainable Development Goal 4 on quality education.

## Figures and Tables

**Table 1:** Characteristics of supplementation intervention trials.

Reference	Study Design	Study Population, Date	Sample Size	Intervention/Duration
**Supplementation of women during pregnancy and lactation**				
Merialdi et al. [33]	RCT	Pregnant women, 10–16 gestational weeks Peru, 1998–2000	242 (1:1)	Zinc + folic acid + iron vs. IFA Daily, 10–16 gestational week until 1 month postpartum
Christian et al. [35]	Cluster RCT	Pregnant and lactating women Nepal, 1999–2001	426 communities, (1:1:1:1:1) 4926 pregnant women.	VA Folic acid + VA IFA + VA Zinc + folic acid + iron + VA MMNs + VA Daily supplementation from 11 (±5.1) gestational week until up to 12 weeks postpartum
Osrin et al. [34]	RCT	Pregnant women, 12–20 gestational weeks. Nepal, 2002–2004	1200 (1:1)	UNIMMAP MMNs vs. IFA Daily supplementation between 12 weeks gestation until childbirth.
Shankar et al. [36]	Cluster RCT	Pregnant women Indonesia, 2001–2004	31,290 (1:1) IFA (15,486) MMN (15,804)	UNIMMAP MMN vs. IFA Daily supplementation between enrolment (34% in 1st trimester, 43% in 2nd trimester, and 23% in 3rd trimester) and 3 months postpartum
Fawzi et al. [30]	RCT	Pregnant women, 12–27 gestational weeks Tanzania, 2001–2005	8428 (1:1) Control arm (4214) Intervention arm (4214)	IFA + MVs vs. IFA + Placebo Daily supplementation from 12–27 gestational weeks to 6 weeks after childbirth
Zeng et al. [37]	Cluster RCT	Pregnant women, 13.8 ± 5.6 gestational weeks China 2002–2006	5828 women enrolled (1:1:1) to one of the three groups. FA (n = 2017), IFA (n = 1912), MMN (n = 1899)	UNIMMAP MMN, IFA, FA Daily supplementation from 13.8 (±5.8) gestational weeks to childbirth.
**Supplementation of infants and young children**				
Tielsch et al. [38,39]	Cluster RCT, 2 × 2 Factorial design	1–36 months of age children Nepal, 2001–2006	426 communities, (1:1:1) A total of 26,250 infants and young children. Placebo (8411), IFA (8128), and IFAZn (8951)	IFAZn vs. IFA vs. Placebo Daily supplementation from 12–35 months of age (length of supplementation depended on age at enrolment)
McDonald et al. [31]	RCT, 2 × 2 Factorial design	Infants, 1 month of age (age at randomization 5.9 ± 0.4 weeks). Tanzania, 2007–2011	2400 infants. Placebo (604), Zn (596), MVs (598), and MVs + Zn (602).	Placebo vs. Zn vs. MVs vs. MVs + Zn Daily supplementation for 18 months. 1–6 months old infants received one dose daily. Infants received two doses daily from 7 months.
Yousafzai et al. [40]	Cluster RCT, 2 × 2 Factorial design	Infants and young children (2–24 months). Pakistan, 2009–2012	1489 infants. Routine health and nutrition services (controls; 368), nutrition education and MNPs (enhanced nutrition; 364), responsive stimulation (responsive stimulation; 383), or a combination of both enriched interventions (374).	MNP vs. No MNP Daily supplementation from (6–24 months).

IFAZn, Iron + Folic acid + Zinc; MMNs, Multiple micronutrients; MNPs, multiple-micronutrient powders; MVs, Multiple vitamins; RCT, Randomized controlled trial; Zn, Zinc; UNIMMAP, United Nations International Multiple Micronutrient Antenatal Preparation; VA, Vitamin A.

**Table 2:** Composition of (daily) micronutrient interventions in studies included in the systematic review [41].

	Vitamin A (µg RAE)	B1 (mg)	B2 (mg)	B3 (mg)	B6 (mg)	B12 (µg)	Folic Acid (µg)	Vit. C (mg)	Vit. D (µg)	Vit. E (mg)	Iron (mg)	Zinc (mg)	Cu (mg)	I (µg)	Se (µg)
**Supplementation of women during pregnancy and lactation—Composition (% RDA [42])**															
Merialdi et al. [33] IFAZn							250 (71)				60 (222)	25 (227)			
Christian et al. [35] IFAZn	1000 (130)						400 (113)				60 (222)	30 (273)			
Christian et al. [35] MMNs ^1^	1000 (130)	1.6 (114)	1.8 (129)	20 (111)	2.2 (116)	2.6 (100)	400 (113)	100 (118)	10 (67)	10 (67)	60 (222)	30 (273)	2.0 (200)		
Osrin et al. [34] UNIMMAP MMNs	800 (104)	1.4 (100)	1.4 (100)	18 (100)	1.9 (100)	2.6 (100)	400 (113)	70 (82)	5.0 (33)	10 (67)	30 (111)	15 (136)	2.0 (200)	150 (68)	65 (108)
**Shankar et al.** [36] **UNIMMAP MMNs**	800 (104)	1.4 (100)	1.4 (100)	18 (100)	1.9 (100)	1.6 (62)	400 (113)	70 (82)	5.0 (33)	10 (67)	30 (111)	15 (136)	2.0 (200)	150 (68)	65 (108)
Fawzi et al. [30] MVs		20 (1429)	20 (1429)	100 (556)	25 (1316)	50 (1923)	800 (227)	500 (588)		30 (200)					
**Zeng et al.** [37] **UNIMMAP MMNs**	800 (104)	1.4 (100)	1.4 (100)	18 (100)	1.9 (100)	2.6 (100)	400 (113)	70 (82)	5.0 (33)	10 (67)	30 (111)	15 (136)	2.0 (200)	150 (68)	65 (108)
**Supplementation of infants and young children**															
Tielsch et al. [38,39] IFAZn							50				12.5	10			
**McDonald et al.** [31] **MVs (+Zn)**		0.5	0.6	4	0.6	1.0	130	60		8.0		5.0			
Yousafzai et al. [40] MNPs ^2^	X						X	X			X				

B1, Thiamine; B2, Riboflavin; B3, Niacin; B6, Pyridoxine; B12, Cobalamin; Cu, Copper; I, Iodine; IFAZn, Iron + Folic acid + Zinc; MMNs, Multiple micronutrients; MNPs, multiple-micronutrient powders; MVs, Multiple vitamins; Se, Selenium; Vit; Zn, Zinc. Additional compositions: ^1^ Christian et al. [43]—vitamin K (65 µg), magnesium (100 mg). ^2^ Composition not reported in the paper. MNP contained iron, folic acid, vitamin A, and vitamin C.

**Table 3:** Risk of bias of the studies included in the systematic review [41].

	Risk of Bias Domains					
Reference	Randomization Process	Intervention Deviations	Missing Outcome Data	Measurement of the Outcome	Selection of the Reported Result	Overall Risk of Bias
Caulfield et al. [46] ^1^	Low	Low	Low	Low	Low	Low
Christian et al. [43] ^2^	Low	Low	Some concerns	Low	Low	Some concerns
Dulal et al. [47] ^3^	Low	Low	Low	Low	Low	Low
Prado et al. [28] ^4^	Low	Low	Low	Low	Low	Low
Sudfeld et al. [44] ^5^	Low	Low	Low	Low	Low	Low
Zhu et al. [48,49] ^6^	Low	Low	Low	Low	Low	Low
Murray-Kolb et al. [43] ^7^	Low	Low	Low	Low	Low	Low
Sudfeld et al. [44] ^8^ Child follow-up	Low	Low	Low	Low	Some concerns	Some concerns
Yousafzai et al. [50] ^9^	Low	Low	Low	Low	Low	Low
Christian et al. [45] ^2,7^	Some concerns	Low	Some concerns	Low	Low	High

Additional references from the parent studies used for the evaluation of RoB: ^1^ Merialdi et al. [32,33]. ^2^ Christian et al. [35]. ^3^ Osrin et al. [34]. ^4^ Shankar et al. [36]. ^5^ Fawzi et al. [30]. ^6^ Zeng et al. [37]. ^7^ Tielsch et al. [38,39]. ^8^ McDonald et al. [31]. ^9^ Yousafzai et al. [40].

**Table 4:** Summary of measures and results of articles included in systematic review [41].

Reference	Study Population, Date	Outcomes	Measures	SMD (95% Confidence Interval)
**Supplementation of women during pregnancy and lactation**				
Caulfield et al. [46] IFAZn vs. IFA	184 children 4–5 years (Peru, 2003–2010)	(1) cognitive development, (3) behavioral development	(1) Wechsler Preschool & Primary Scale of Intelligence	−0.04 (95%CI: −0.33 to 0.25)
			(1) Language development, bear story	0.02 (95%CI: −0.27 to 0.32)
			(1) Number concepts, counting game	0.02 (95%CI: −0.28 to 0.32)
			(1) Goodenough & Harris Draw-a-Person Test	−0.11 (95%CI: −0.42 to 0.19)
			(1) Interpersonal understanding, friendship interview	−0.16 (95%CI: −0.47 to 0.15)
			(3) Vineland Adaptive Behaviour Scales Communication Daily living skills Socialization Motor skills	−0.11 (95%CI: −0.40 to 0.18) 0.06 (95%CI: −0.23 to 0.35) 0.06 (95%CI: −0.23 to 0.36) 0.04 (95%CI: −0.25 to 0.34)
			(3) Preschool Behaviour Questionnaire Internalizing Externalizing	0.13 (95%CI: −0.16 to 0.42) 0.06 (95%CI: −0.23 to 0.35)
Christian et al. [43] ^1^ IFAZn vs. IFA	281 children 7–9 years (Nepal, 2007–2009)	(1) cognitive development, (2) motor development	(1) The Universal Non-Verbal Intelligence Test (UNIT)	−0.17 (95%CI: −0.41 to 0.08)
			(1) Executive function *Go/No-go* test Stroop test(proportion who failed) Backward digit span	**−0.22 (95%CI: −0.46 to 0.03)** **0.33 (95%CI: 0.09 to 0.57)** **−0.33 (95%CI: −0.57 to −0.08)**
			(2) The Movement Assessment Battery for Children (MABC) ^3^	**0.33 (95%CI: 0.08 to 0.57)**
			(2) Finger-tapping test	**−0.41 (95%CI: −0.66 to −0.17)**
Christian et al. [43] ^2^ MMNs vs. IFA	321 children 7–9 years (Nepal, 2007–2009)	(1) cognitive development, (2) motor development	(1) The Universal Non-Verbal Intelligence Test (UNIT)	**−0.26 (95%CI: −0.49 to −0.02)**
			(1) Executive function *Go/No-go* test Stroop test(proportion who failed) Backward digit span	−0.00 (95%CI: −0.24 to 0.23) 0.20 (95%CI: −0.03 to 0.44) **−0.36 (95%CI: −0.60 to −0.13)**
			(2) The Movement Assessment Battery for Children (MABC) ^3^	**0.32 (95%CI: 0.09 to 0.56)**
			(2)Finger-tapping test	**−0.45 (95%CI: −0.69 to −0.22)**
Dulal et al. [47] MMNs vs. IFA	813 young adolescents 12 years (Nepal, 2015–2016)	(1) cognitive development	(1) The Universal Non-Verbal Intelligence Test (UNIT)	0.09 (95%CI: −0.05 to 0.23)
			(1) Executive function using a counting Stroop test	0.10 (95%CI: −0.04 to 0.24)
Prado et al. [28] MMNs vs. IFA	2879 children and young adolescents 9–12 years (Indonesia, 2012–2014)	(1) cognitive development, (2) motor development, (3) behavioral development	(1) General intellectual ability (1) Declarative memory (1) Procedural memory (1) Executive function (1) Academic achievement	0.09 (95%CI: −0.03 to 0.22) 0.01 (95%CI: −0.09 to 0.11) **0.11 (95%CI: 0.01 to 0.20)** 0.07 (95%CI: −0.04 to 0.19) 0.08 (95%CI: −0.05 to 0.21)
			(2) Fine motor dexterity	−0.07 (95%CI: −0.16 to 0.02)
			(3) Socio-emotional health	0.06 (95%CI: −0.04 to 0.16)
Sudfeld et al. [44] IFA + MVs vs. IFA+ Placebo	446 young adolescents 11–14 years (Tanzania, 2015–2017)	(1) cognitive development, (3) behavior development	(1) General Intelligence (Atlantis, Footsteps, Hand movement, Kilifi naming test, Koh’s block design test, Story completion, and verbal fluency)	−0.02 (95%CI: −0.20 to 0.17)
			(1) Executive function (Literacy, Numeracy, *Go/No-go*, People search, ROCF copy, ROCF recall, and Shift)	0.00 (95%CI: −0.19 to 0.19)
			(3) Mental health.(SDQ and the Behaviour Rating Inventory of Executive Function (BRIEF) to assess mental health)	0.05 (95%CI: −0.14 to 0.23)
Zhu et al. [48,49] MMN vs. IFA	1385 children and young adolescents 10–14 years (China, 2016)	(1) cognitive development, (3) behavioral development	(1) Adolescent full-scale intelligence quotient and aspects of verbal comprehension, working memory, perceptual reasoning, and processing speed indexes were assessed by the Wechsler Intelligence Scale for Children	**0.13 (95%CI: 0.03 to 0.24)**
			(3) Internalizing, externalizing, and total behavior problem scores	0.05 (95%CI: −0.06 to 0.16)
**Supplementation of infants and young children**				
Murray-Kolb et al. [51] IFAZn vs. Placebo	377 children 7–9 years (Nepal, 2007–2009)	(1) cognitive development, (2) motor development	(1) The Universal Non-Verbal Intelligence Test (UNIT)	0.11 (95%CI: −0.10 to 0.31)
			(1) Stroop test (proportion who failed)	**−0.29 (95%CI: −0.50 to −0.09)**
			(1) Backward digit span	0.18 (95%CI: −0.02 to 0.39)
			(1) *Go/No-go* test	−0.13 (95%CI: −0.34 to 0.07)
			(2) The Movement Assessment Battery for Children (MABC) ^3^	−0.12 (95%CI: −0.32 to 0.08)
			(2) Finger-tapping	0.18 (95%CI: −0.02 to 0.39)
Sudfeld et al. [44] MVs vs. No MVs	365 children 6–8 years (Tanzania, 2015–2017)	(1) cognitive development, (3) behavioral development	(1) General Intelligence (Atlantis, Footsteps, Hand movement, Kilifi naming test, Koh’s block design test, Story completion, and verbal fluency)	0.00 (95%CI: −0.21 to 0.21)
			(1) Executive function (Literacy, Numeracy, Go/*No-Go*, People search, ROCF copy, ROCF recall, and Shift)	0.00 (95%CI: −0.21 to 0.21)
			(3) Mental health.(SDQ and the Behaviour Rating Inventory of Executive Function (BRIEF) to assess mental health)	0.08 (95%CI: −0.10 to 0.26)
Yousafzai et al. [50] MNP vs. No MNP	1302 children 4 years (Pakistan, 2013)	(1) cognitive development, (2) motor development, (3) behavioral development	(1) Cognitive capacity including Intelligent quotient Executive function Pre-academic skills	−0.10 (95%CI: −0.21 to 0.02) −0.03 (95%CI: −0.15 to 0.09) **0.16 (95%CI: 0.05 to 0.27)**
			(2) Motor development	0.11 (95%CI: −0.01 to 0.24)
			(3) Social-emotional development Pro-social behaviors Behavioral problems	−0.09 (95%CI: −0.20 to 0.01) −0.02 (95%CI: −0.13 to 0.09)
**Supplementation of women during pregnancy and lactation and of infants and young children**				
Christian et al. [45] M-IFAZn C-IFAZn vs. M-IFA C-Pl	223 children 7–9 years (Nepal, 2007–2009)	(1) cognitive development, (2) motor development	(1) The Universal Non-Verbal Intelligence Test (UNIT)	−0.16 (95%CI: −0.43 to 0.10)
			(1) Stroop test (proportion who failed)	**0.40 (95%CI: 0.13 to 0.66)**
			(1) Backward digit span	**−0.44 (95%CI: −0.71 to −0.18)**
			(1)*Go/No-go* test	−0.22 (95%CI: −0.48 to 0.05)
			(2) The Movement Assessment Battery for Children (MABC) ^3^	**0.34 (95%CI: 0.07 to 0.61)**
			(2) Finger-tapping	**−0.46 (95%CI: −0.72 to −0.19)**

C-IFAZn, Child IFAZn; C-Pl, Child Placebo; IFAZn, Iron + Folic acid + Zinc; M-IFA, Maternal IFA; M-IFAZn, Maternal IFAZn; MMNs, Multiple micronutrients; MNP multiple-micronutrient powder; MVs, Multiple vitamins; *Go/No go* test for sustained attention and response control; ROCF, Rey–Osterrieth complex figure; SDQ, Strengths and Difficulties Questionnaire; SMD, Standard mean difference; Zn, Zinc; ^1^ Comparison between the effect of IFAZn and IFA; ^2^ Comparison between the effects of MMNs and IFA; ^3^ Higher scores are worse outcomes. Values shown in bold indicate statistical significance.

**Table 5:** Effect direction plot of the long-term effects of MMN supplementation on cognitive development. Study design; RCT: Randomized Controlled Trial; cRCT: Cluster Randomized Trial; Effect direction; upward arrow ▲ = positive impact, downward arrow ▼ = negative impact, sideways arrow ◄► = no change/mixed effects/conflicting findings; Study quality: denoted by row color: green = low risk of bias; amber = some concerns; red = high risk of bias. Sudfeld 2019 [44], reported on two interventions in Tanzania.

Study	Study Design	Cognitive Development	Motor Development	Behavioral Development
Christian et al. [43]	cRCT	◄►	▼	
Sudfeld et al. [44]	RCT	◄►		◄►
Murray-Kolb et al. [43]	cRCT	◄►	◄►	
Caulfield et al. [46]	RCT	◄►		◄►
Yousafzai et al. [50]	cRCT	◄►	◄►	◄►
Prado et al. [28]	cRCT	◄►	◄►	◄►
Dulal et al. [47]	RCT	◄►		
Zhu et al. [48,49]	cRCT	▲		◄►
Sudfeld et al. [44]	RCT	◄►		◄►
Christian et al. [45]	cRCT	◄►	▼	

## Data Availability

No new data were created or analyzed in this study. Data sharing is not applicable to this article.

## Supplementary Materials

The following supporting information can be downloaded at https://www.mdpi.com/article/10.3390/nu17243966/s1: Table S1. PRISMA 2020 Checklist. Table S2. Search strategy. Table S3. Standardized tools and tests for assessing cognitive development.
