# Supplementary material for: Long-Term Effects of Multiple-Micronutrient Supplementation During Pregnancy, Lactation, and Early Childhood on the Cognitive Development of Children Aged 4–14 Years: A Systematic Review of Randomized Controlled Trials
Source: Nutrients. 2025 Dec 18;17(24):3966. doi: 10.3390/nu17243966 (PMC12736284; doi:10.3390/nu17243966)
Supplement: Supplementary file 1 [file nutrients-17-03966-s001.zip › nutrients-3988200-supplementary.pdf]

SUPPLEMENTARY MATERIALS

Table S1. PRISMA 2020 Checklist.

Section and Topic	Item #	Checklist item	Location where item is reported
**TITLE**			
**Title**	1	Identify the report as a systematic review.	Title
**ABSTRACT**			
**Abstract**	2	See the PRISMA 2020 for Abstracts.	We reviewed the checklist and applied it.
**INTRODUCTION**			
**Rationale**	3	Describe the rationale for the review in the context of existing knowledge.	Introduction
**Objectives**	4	Provide an explicit statement of the objective(s) or question(s) the review addresses.	Introduction
**METHODS**			
**Eligibility criteria**	5	Specify the inclusion and exclusion criteria for the review and how studies were grouped for the syntheses.	Data sources and search strategy<br>Eligibility criteria
**Information sources**	6	Specify all databases, registers, websites, organisations, reference lists and other sources searched or consulted to identify studies. Specify the date when each source was last searched or consulted.	Method and design<br>Data sources and search strategy
**Search strategy**	7	Present the full search strategies for all databases, registers and websites, including any filters and limits used.	Annex 2
**Selection process**	8	Specify the methods used to decide whether a study met the inclusion criteria of the review, including how many reviewers screened each record and each report retrieved, whether they worked independently, and if applicable, details of automation tools used in the process.	Eligibility criteria<br>Study selection
**Data collection process**	9	Specify the methods used to collect data from reports, including how many reviewers collected data from each report, whether they worked independently, any processes for obtaining or confirming data from study investigators, and if applicable, details of automation tools used in the process.	Data extraction<br>Data Synthesis
**Data items**	10a	List and define all outcomes for which data were sought. Specify whether all results that were compatible with each outcome domain in each study were sought (e.g. for all measures, time points, analyses), and if not, the methods used to decide which results to collect.	Data extraction<br>Data Synthesis
	10b	List and define all other variables for which data were sought (e.g. participant and intervention characteristics, funding sources). Describe any assumptions made about any missing or unclear information.	Data extraction<br>Data Synthesis
**Study risk of bias assessment**	11	Specify the methods used to assess risk of bias in the included studies, including details of the tool(s) used, how many reviewers assessed each study and whether they worked independently, and if	Assessment of risk of bias

		applicable, details of automation tools used in the process.	
**Effect measures**	12	Specify for each outcome the effect measure(s) (e.g. risk ratio, mean difference) used in the synthesis or presentation of results.	Data Synthesis<br>Analysis of subgroups
**Synthesis methods**	13a	Describe the processes used to decide which studies were eligible for each synthesis (e.g. tabulating the study intervention characteristics and comparing against the planned groups for each synthesis (item #5)).	Data Synthesis
	13b	Describe any methods required to prepare the data for presentation or synthesis, such as handling of missing summary statistics, or data conversions.	Data Synthesis
	13c	Describe any methods used to tabulate or visually display results of individual studies and syntheses.	
	13d	Describe any methods used to synthesize results and provide a rationale for the choice(s). If meta-analysis was performed, describe the model(s), method(s) to identify the presence and extent of statistical heterogeneity, and software package(s) used.	
	13e	Describe any methods used to explore possible causes of heterogeneity among study results (e.g. subgroup analysis, meta-regression).	
	13f	Describe any sensitivity analyses conducted to assess robustness of the synthesized results.	
**Reporting bias assessment**	14	Describe any methods used to assess risk of bias due to missing results in a synthesis (arising from reporting biases).	Assessment of risk of bias
**Certainty assessment**	15	Describe any methods used to assess certainty (or confidence) in the body of evidence for an outcome.	
**RESULTS**			
**Study selection**	16a	Describe the results of the search and selection process, from the number of records identified in the search to the number of studies included in the review, ideally using a flow diagram.	Result<br>Fig. 1
	16b	Cite studies that might appear to meet the inclusion criteria, but which were excluded, and explain why they were excluded.	
**Study characteristics**	17	Cite each included study and present its characteristics.	Study characteristics
**Risk of bias in studies**	18	Present assessments of risk of bias for each included study.	Risk of bias
**Results of individual studies**	19	For all outcomes, present, for each study: (a) summary statistics for each group (where appropriate) and (b) an effect estimate and its precision (e.g. confidence/credible interval), ideally using structured tables or plots.	Results
**Results of syntheses**	20a	For each synthesis, briefly summarise the characteristics and risk of bias among contributing studies.	Risk of bias
	20b	Present results of all statistical syntheses conducted. If meta-analysis was done, present for each the summary estimate and its precision (e.g. confidence/credible interval) and measures of	Results

		statistical heterogeneity. If comparing groups, describe the direction of the effect.	
	20c	Present results of all investigations of possible causes of heterogeneity among study results.	Results
	20d	Present results of all sensitivity analyses conducted to assess the robustness of the synthesized results.	Results
**Reporting biases**	21	Present assessments of risk of bias due to missing results (arising from reporting biases) for each synthesis assessed.	Results
**Certainty of evidence**	22	Present assessments of certainty (or confidence) in the body of evidence for each outcome assessed.	Results
**DISCUSSION**			
**Discussion**	23a	Provide a general interpretation of the results in the context of other evidence.	Discussion
	23b	Discuss any limitations of the evidence included in the review.	Discussion
	23c	Discuss any limitations of the review processes used.	
	23d	Discuss implications of the results for practice, policy, and future research.	Conclusion
**OTHER INFORMATION**			
**Registration and protocol**	24a	Provide registration information for the review, including register name and registration number, or state that the review was not registered.	Methods and design
	24b	Indicate where the review protocol can be accessed, or state that a protocol was not prepared.	Methods and design
	24c	Describe and explain any amendments to information provided at registration or in the protocol.	
**Support**	25	Describe sources of financial or non-financial support for the review, and the role of the funders or sponsors in the review.	
**Competing interests**	26	Declare any competing interests of review authors.	
**Availability of data, code and other materials**	27	Report which of the following are publicly available and where they can be found: template data collection forms; data extracted from included studies; data used for all analyses; analytic code; any other materials used in the review.	Data Availability section, Data will be fully available under request to the corresponding author

Table S2. Search strategy

Research question (e.g., PICO format): What are the long-term impacts of micronutrient supplementation of children and pregnant and lactating women on the children cognitive development?			
**NAME OF DATABASE (interface): List of terms for the search strategy**			
**Concept**	**Line number**	**Search strategy**	
**Concept 1: Cognition**	1	1. Cognition<br>2. Cognitive development<br>3. Cognitive neuroscience<br>4. Child development<br>5. Language development<br>6. Intelligence tests<br>7. Intelligence quotient<br>8. Neuropsychological test<br>9. Wechsler scales[1]<br>10. Stanford Binet test[2]<br>11. Developmental psychology<br>12. Academic achievement	13. Academic performance<br>14. Learning curve<br>15. Aptitude test[3]<br>16. Multitasking behaviour<br>17. Executive function<br>18. Learning<br>19. Problem solving<br>20. Thinking<br>21. social-emotional development<br>22. Verbal skills/verbal behavior<br>23. Adaptive skills
**Concept 2: Infants, toddlers and children**	2	1. Infant<br>2. Toddler<br>3. Preschool child<br>4. Child<br>5. School child	6. Student<br>7. Pupil<br>8. School<br>9. Elementary student<br>10. Primary school
**Concept 3: Pregnancy and lactation**	3	1. Prenatal/Antenatal<br>2. Lactation/breastfeeding<br>3. Pregnancy	4. Postnatal/postpartum<br>5. Perinatal

[1] Tests designed to measure intellectual functioning in children and adults.
[2] An individual intelligence test designed primarily for school children to predict school performance and the ability to adjust to everyday demands.
[3] aptitude tests are used to measure the potential ability to learn.

**Concept 4: Micronutrient Supplementation**	4	1. Micronutrient supplementation<br>2. Multiple micronutrient powder<br>3. Dietary Supplements<br>4. Diet supplementation<br>5. Mineral supplementation<br>6. Zinc<br>7. Iron	8. Iodine<br>9. Folic acid<br>10. B12<br>11. Vitamin A<br>12. Vitamin D<br>13. Calcium<br>14. Vitamin C
**Concept 5: Study design**	5	1. Clinical Trial<br>2. Randomized Controlled Trial<br>3. Controlled trial	4. Quasi-experimental trial<br>5. Prospective cohort study<br>6. Retrospective cohort study<br>7. Repeated cross-sectional
**Concept 7: Low and middle income countries**	6	1. Countries/names<br>2. Developing countries<br>3. Low and middle income countries	
**Combination of concepts**	7	1 AND (2 OR 3) AND 4 AND 5 AND 6	

Table S3. Standardized tools and tests for assessing cognitive development.

Cognitive domain / Sub-domain	Test	Description
**General intellectual ability**		
Verbal ability: general knowledge	Information test	Children were required to answer general knowledge questions verbally, such as “How many days are in a week?” The score was the number of questions answered correctly.
verbal comprehension, perceptual reasoning, working memory, and processing speed	Wechsler Intelligence Scale for Children	It is linked to brain impairment and its implication on adaptive functioning. It assesses intellectual abilities across verbal comprehension, perceptual reasoning, working memory, and processing speed. The test is suitable for children between 6–16 years [41, 42].
Language development	Language development: Bear story	Test to obtain indications of the child’s language development and narrative ability. It is assessed using a rating scale from 1-11. This subdomain is within the Wechsler Intelligence Scale (WISC IV) [43, 44].
IQ performance (Children's numbers concepts)	Number concepts: the counting game	The test is used to assess three subdomains within general intelligence ability; Counting, one-to-one correspondence, and the ability to integrate two dimensions)
Concept formation: draw a person	Goodenough & Harris Draw-a-Person Test	This test measures the complexity of the children’s concept formation ability. Its scoring system indicated the presence or absence of specific components (e.g., head, hair, eyes, trunk, legs, and arms)
Socio cognitive	Interpersonal understanding, friendship interview	It assesses the child’s knowledge of friendship concepts within the context of the child’s friends [38].
Adaptive behaviors	Vineland Adaptive Behavior Scales	The Vineland Adaptive Behavior Scales measure communication, daily living, socialization, and motor skills. It is suitable for assessing individuals from 0–90 years of age. It assesses four subdomains Communication, Daily Living, Socialization, and Motor skills [45, 44]
Behavioral development	Preschool Behavior Questionnaire	Preschool Behavior Questionnaire, is a 30-item instrument designed to assess the child’s behavior problems. This tool is intended for preschool children and is suitable for children aged 3–6 years [50].
Verbal ability: semantic memory and lexical retrieval	Speeded picture naming test	Children were instructed to point to and say out loud the name of each picture on a page as quickly and accurately as possible. The score was calculated as the time to complete the page divided by the number of pictures correctly named.
Non-verbal ability: spatial	Block design test	Children were asked to copy increasingly complex patterns with colored blocks. The score takes into account both

pattern copying		accuracy and speed.
Memory and reasoning, Symbolic	Universal Non-verbal Intelligence Test (UNIT)	They are designed to measure cognitive abilities. It is used with individuals aged 5 years and older and applicable across a wide range of age groups, from children to adults. The tool uses nonverbal stimuli and requires minimal verbal communication, making it suitable for individuals with language barriers, hearing impairments, or speech-language disorders [44].
Other test for general intelligence ability	Atlantis, Footsteps, Hand movement, Kilifi naming test, Koh's block design test, Story completion, and verbal fluency)	These tests are a subset of the general intelligence ability. They measure general intellectual function and assess a broad range of functioning through multiple subtests [51]
**Declarative memory**		
Declarative memory	Adapted Rey auditory verbal learning test	Children were given three learning trials in which they were asked to remember a list of 11 unrelated words presented orally. This test was followed by an interference trial requiring the immediate recall of a second 11-word list, and then a request to recall the first list (recall trial 1). After a delay of a mean of 7 min, participants were again asked to recall the initial list (recall trial 2) and then given a recognition test.
**Procedural memory**		
Procedural memory	Serial reaction time task	Children did the task with a video game pad controller and a laptop. Children were required to press the button on the gamepad that corresponded to the position on the screen in which a smiley face appeared. A random block (of 60 items) was followed by four blocks that presented a standard ten-item sequence, followed by a final random block. The procedural learning score was the difference between the mean standardized reaction time on the final random block and the fourth sequence block.
**Executive function**	Behavior Rating Inventory of Executive Function (BRIEF)	Observational tools are used to evaluate executive functions such as inhibitory control, working memory, emotional control, and planning/organizing [42].
Visual attention	Adapted visual search task	Based on the Sky Search subtest from the Test of Everyday Attention for Children (TEACh), a local illustrator drew a series of pairs of pictures, some of which were the same and some of which were different. Children were asked to underline all pairs that were the same as fast as possible. The score was the time per correct target on the visual search task minus the time per correct target on a motor control task.
Sustained attention	Adapted visual search dual-task	Based on the Sky Search Dual Task subtest from the TEACh, children were asked to complete a parallel version of the visual search task described above, which differs only in the location of the targets. As they did the visual search task, they were asked to simultaneously and silently count the number of tones presented in each item of a tone-counting task. The score takes into account performance on both tasks.

Auditory attention and working memory	Digit span forward and backward	The digit span forward and backward scores were calculated as the total number of sequences of digits, correctly repeated (digit span forward) or repeated in reverse order (digit span backward), before an error was committed on two consecutive trials of the same length.
Cognitive control	Stroop test numbers	Children were presented with four conditions, each consisting of 20 items. The first and last were baseline conditions, consisting of zeros (000), where children were required to name the number of zeros in each item (three, four, five, or six). The second was a congruent condition where the quantity corresponded to the printed number (e.g., 333). The third was an incongruent condition where the quantity and the printed number did not correspond (e.g., 222). Again, the task was to name the quantity, not the printed number. The total time to correctly name all of the items in each condition was recorded. The interference score was calculated as the time to complete the incongruent condition minus the time to complete the congruent condition.
Cognitive control / Inhibitory control.	Go/No-go test	This test is a subdomain of the executive functioning of the child. It assesses the ability of the children's cognitively to inhibit and control their impulsive actions and performance as cognitive load increases. It also explores the attention capacity [52]
Cognitive flexibility	NIH Toolbox Dimensional Change Card Sort Test	We used the e-Prime version. Children were shown pictures on a tablet screen, which differed on two characteristics: shape (a truck or a ball) and color (blue or yellow). In each trial, children were instructed to match the picture at the top of the screen to the picture on the right or the left according to the verbal computerized instructions (shape or color). We calculated the score according to the standard National Institute of Health Toolbox method.
**Educational attainment**		
Literacy	Literacy test	Children were given a letter discrimination task, a word discrimination task, and a sentence discrimination task. They were instructed to mark real letters, real words, and sentences that were answered "yes" (Do birds have wings?) but not those answered "no" (Do cars have feet?) The score was the sum of the hits (correctly marked) minus false alarms (incorrectly marked) with additional points given for faster performance on the sentence task.
Arithmetic	Arithmetic test	Children were verbally asked arithmetic questions and required to answer without doing written calculations. We developed a set of items from elementary school arithmetic textbooks. The score was the total number correct.
**Fine motor**		
Motor dexterity	Purdue pegboard test	We recorded the number of pegs children were able to place on a board in the 30s, first with the right hand, then with the left hand, and then with both hands simultaneously. The pegboard average score was the average of these three trials. In the assembly trial, the child was required to assemble a peg, a washer, a collar, and another washer in each hole on the board. The pegboard assembly score was the number of pieces correctly assembled.

Motor skills	Movement Assessment Battery for Children (MABC-2)	It has domains such as attention and concentration, problem solving, planning, and organization which are designed to assess motor function of children aged 3–16 years (categorized into three age bands 3–6, 7–10, and 11–16 years)
Complex motor skills	Finger-tapping test	This test is employed to measure motor performance, especially in the upper extremities of the body [53].
**Socio-emotional**		
Behavioral problems	Adapted Child Behavior Checklist	We developed a 50-question interview representing seven subscales of the checklist: depression, social problems, thought problems, attention problems, delinquent behavior, aggressive behavior, and other problems. The total score was the sum of the item scores.
